# Revealing the antipolar order in the antiferroelectric SmZ_A_ phase by means of circular alignment

**DOI:** 10.1038/s41598-024-65275-y

**Published:** 2024-07-01

**Authors:** Pierre Nacke, Rachel Tuffin, Melanie Klasen-Memmer, Per Rudquist, Frank Giesselmann

**Affiliations:** 1 Institute of Physical Chemistry, University of Stuttgart, 70569 Stuttgart, Germany; 2 Display Solutions, Merck Electronics KGaA, 64293 Darmstadt, Germany; 3 Department of Microtechnology and Nanoscience, Chalmers University of Technology, 41296 Gothenburg, Sweden

**Keywords:** Soft materials, Liquid crystals, Ferroelectrics and multiferroics

## Abstract

Many ferroelectric nematic liquid crystals, like one of the archetype materials, DIO, do not have a direct paraelectric N to ferroelectric N_F_ phase transition, but exhibit yet another phase between N and N_F_. This phase has recently been proposed to be antiferroelectric, with a layered structure of alternating polarization normal to the average director and is sometimes referred to as Smectic Z_A_ (SmZ_A_). We have examined the SmZ_A_ phase in circularly rubbed (CR) cells, known to discriminate between the polar N_F_ and the non-polar N phase from the configuration of disclination lines formed. We find that the ground state of SmZ_A_ has the same disclination configuration as the non-polar N phase, demonstrating that the SmZ_A_ phase is also non-polar, i.e., it has no net ferroelectric polarization. At the same time, the SmZ_A_ texture generally has a grainy appearance, which we suggest is partly a result of the frustration related to layered order combined with the imposed twist in CR cells. We discuss possible orientations of the smectic layers, depending on the alignment conditions. While a horizontal SmZ_A_ layer structure is always compatible with surface-induced twist, a vertical layer structure would tend to break up in a twisted bookshelf structure to match non-parallel alignment directions at the two surfaces.

## Introduction

**Figure 1:**
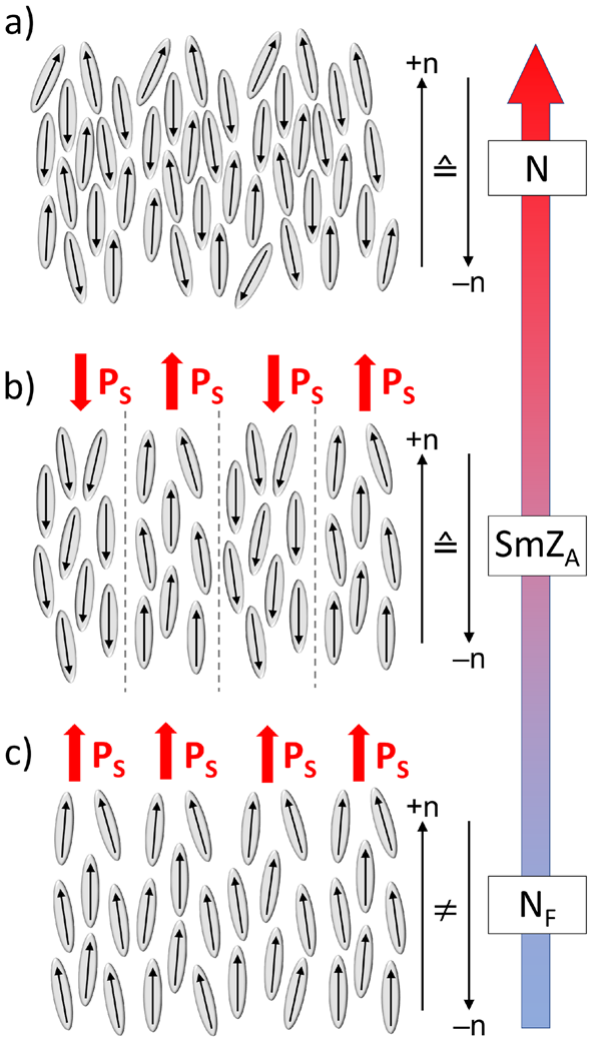
Schematics of (**a**) the nematic phase N, (**b**) the antiferroelectric smectic Z_A_ phase SmZ_A_ and (**c**) the ferroelectric nematic phase N_F_. The details of the polarization inversion regions in SmZ_A_ (marked with dashed lines) are not addressed in this study. From reference^44^ used with permission.

The simplest form of liquid crystal—the nematic phase N—is a three-dimensional uniaxial anisotropic fluid, oriented along the local symmetry axis described with the director **n**. N is non-polar, which means that **n** is invariant under sign inversion, i.e., +**n** = –**n**, cf. Fig. 1a. In 2017, Mandle et al.^1^ and Nishikawa et al^2^ reported liquid crystals of highly polar molecules (RM734 and DIO, respectively) that seemed to exhibit more than one nematic phase, some of which appeared to defy the sign-invariance of n. This means that they ought to be, at least locally, spontaneously polar. Later Chen et al. presented evidence^3^ that RM734, originally synthesized and studied by Mandle^1,4–6^, indeed exhibits a ferroelectric nematic phase (N_F_) with a spontaneous electric polarization **P**, cf. Fig. 1b. The N_F_ phase of RM734 is perfectly miscible with, and therefore also identical to, the lowest temperature range nematic phase in DIO^3,7^. Ferroelectric nematics today constitute an intense field of research^1–41^ (so far about 150 N_F_ materials have been reported^23,42,43^) but the mechanisms behind spontaneously polarized nematics are still not fully understood. Especially, this holds true for the additional phase, originally called M2, located between the N_F_ phase and the N phase in the archetype N_F_ material DIO^2^.

The structure and polar nature of this M2 phase have since been studied intensely by several groups^8,28,35,45^. In 2021, at the ferroelectric liquid crystal conference in Ljubljana, Slovenia, Clark reported evidence that this phase in fact has antiferroelectric order, with “layers” of alternating polarization directions (Fig. 1c)^46^. The periodicity of the layered phase was revealed by the electron density variation between splayed and non-splayed regions probed using synchrotron based small angle X-ray scattering (SAXS) by Clark et al.^45^ and later by Cruichshank et al.^35^. The period of the alternating polarization field is two times the period of the electron density modulation. As the phase is “layered” it was pointed out that it is in fact a smectic material and the phase was coined Smectic  Z_A_  (SmZ_A_)^45,46^. The SAXS experiments further showed that the “layer thickness” in SmZ_A_ of DIO is about 10 nm, corresponding to about 20 molecular widths. In comparison, in the N_F_  phase ferroelectric syn-polar domains span several micrometers^6^. Importantly, over multiple layers the antipolar arrangement of polar nematic substructures, restores a sign inversion invariance in the SmZ_A_ phase and there should be no global ferroelectric polarization. This was confirmed via dielectric measurements by Brown et al.^8^ and Erkoreka et al.^47^. Interestingly, the antiferroelectric structure of SmZ_A_ might mediate the transition from the conventional paraelectric nematic to the ferroelectric nematic phase.

**Figure 2:**
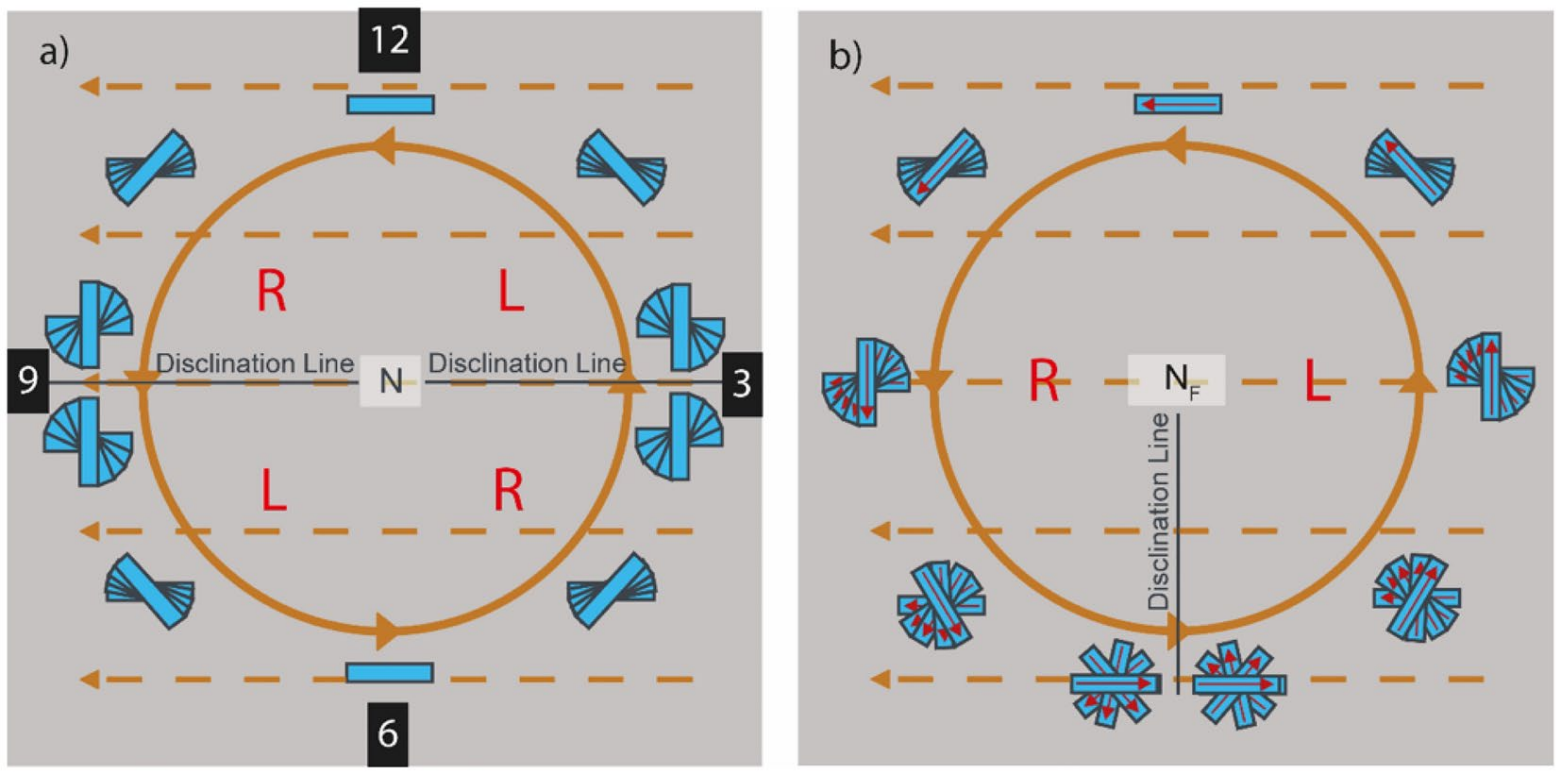
Schematic drawing of the ordinary nematic phase (**a**) and the ferroelectric nematic phase (**b**) in so-called circularly rubbed cells^15^. These cells have two distinctive alignment layers. One at the top plate (solid brown lines), which is rubbed in a circular fashion and one at the bottom (dotted brown lines), where the rubbing direction is linear. Given that the director **n** follows the rubbing direction at the surfaces, a characteristic disclination pattern emerges. The regular non-polar nematic (**a**) displays two disclination lines that run parallel to the linear rubbing. In contrast, the polar ferroelectric nematic (**b**) displays a single line that runs perpendicular to the linear rubbing direction. L (left) and R (right) denote the handedness of the twist in that region.

Conventional rubbed polyimide surfaces provide in-plane polar anchoring of the N_F_ phase^10,15^. In sandwich cells with antiparallel rubbing on the two surfaces, the N_F_ therefore tends to form ± 180° twisted states, with the same probability for right-, and left-handed twist. This is in stark contrast to the non-polar N phase, where no twist occurs for antiparallel nor for parallel rubbing conditions. If the two surfaces are rubbed in a linear, and a circular fashion, respectively (circularly rubbed cells, CR cells), the N and N_F_, or rather nonpolar and polar nematics, can easily be distinguished from the characteristic disclination lines formed, cf. Fig. 2^15^.

The CR cell-method for identification of N and N_F_ phases is described in ref.^15^ and in Fig. 2. In short, in a CR cell the conventional N makes two disclination lines, from the center of circular rubbing along the two radii where the mutual rubbing directions are orthogonal (position 9 and 3 on a clock face). At these disclinations the director twist jumps from + 90° to −90° to minimize the twist elastic energy. Everywhere else in the CR cell, there is a continuous twist with different magnitude between the surfaces. In the N_F_ phase, due to the in-plane polar surface anchoring and the bulk polar order of the phase, there is instead one disclination line, in the direction where the mutual rubbing directions are antiparallel (position 6). At this single disclination line the twist jumps from + 180° to −180°.

Here we use the CR cell-method to study the polar nature of the SmZ_A_ phase in DIO. We find that the ground state of SmZ_A_ has the same two-line defect configuration as the conventional N phase, in agreement with the proposed macroscopically non-polar structure of SmZ_A_. Furthermore, the CR cell geometry gives information about the orientation of the SmZ_A_ layers in relation to the mutual rubbing directions, as all combinations of rubbing angles are represented in the same CR cell. Parallel as well as and antiparallel rubbing directions allow for the layers to form normal to (bookshelf orientation) or parallel (planar orientation) to the surfaces. Non-parallel rubbing, on the other hand, is obviously incompatible with a bookshelf layer structure (cf. SI Fig. S1), and should instead promote planar orientation of the layers, which inherently allows for a director twist normal to the plates, or force the SmZ_A_ structure to break up into a broken helix structure of azimuthally shifted blocks of SmZ_A_. (SI Fig. S2). This would constitute a twisted bookshelf comparable to a twist grain boundary (TGB)-like structure known from some chiral smectics^48^.

## Results and discussion

**Figure 3:**
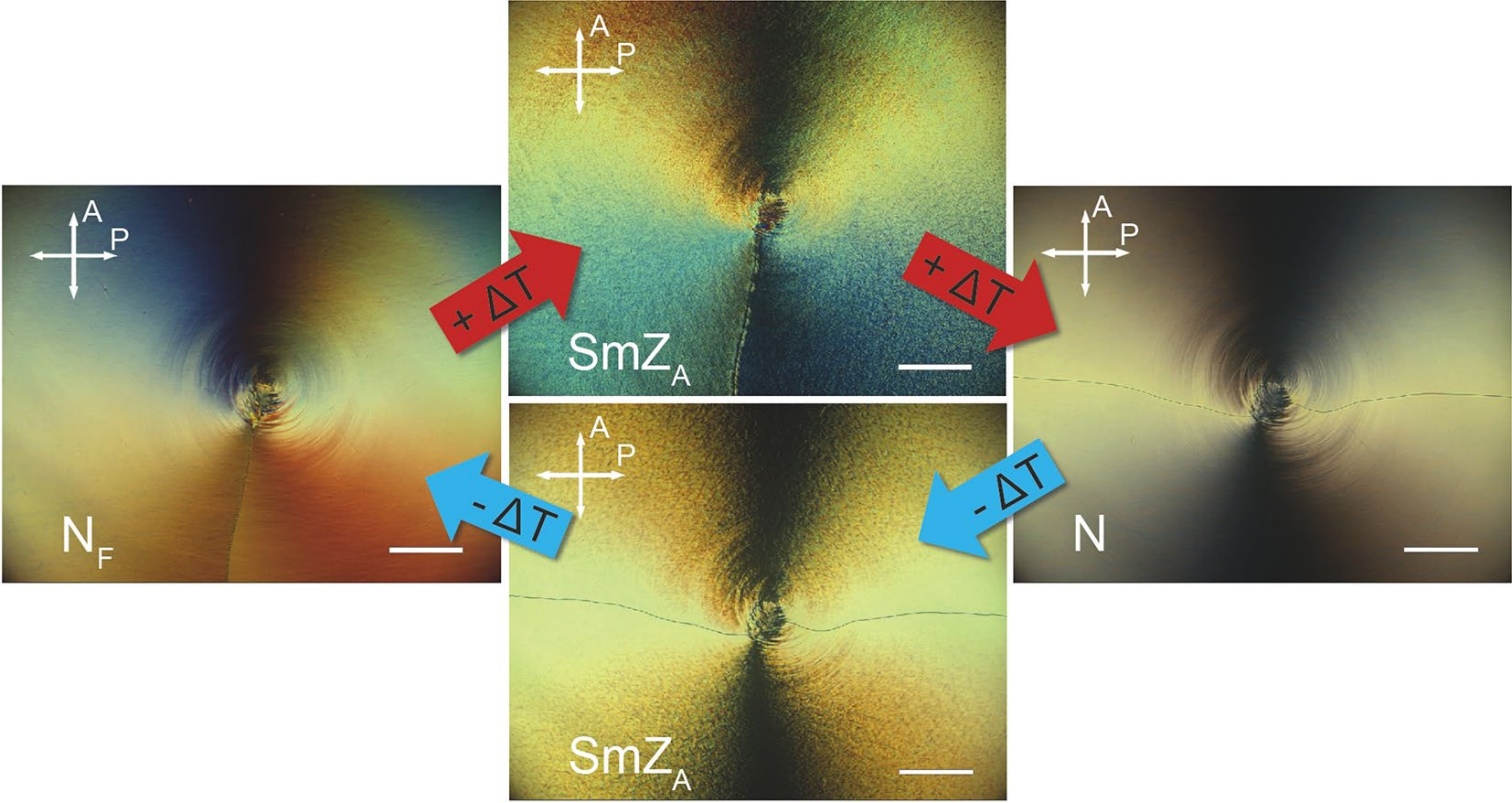
Polarized light microscopy photographs of DIO in a circularly rubbed cell in the ordinary nematic N, the SmZ_A_ and the ferroelectric nematic N_F_ phase. Starting with the nematic phase (picture at 120 °C) and cooling down to SmZ_A_ (picture at 82 °C) the texture shows two disclination lines. On further cooling, these merge into one single line in the N_F_ phase (picture at 60 °C). When heating up again into the SmZ_A_ phase (picture at 75 °C) the disclination pattern of the N_F_ phase is kept (metastable state). The line splits again in two after heating into the nematic phase. (scale bars: 200 µm).

Figure 3 shows a 4 µm thick CR cell filled with DIO between crossed polarizers in the optical microscope. The cell was first cooled down from the isotropic phase at a rate of 1 K/min. At the transition to N (*T* = 172 °C) a characteristic smooth texture with two disclination lines at 9 and at 3 o'clock was immediately produced (right). On further cooling at 1 K/min (Fig. 3, bottom path) the texture changed at *T* = 83 °C to a microdomain, grainy texture, with a characteristic domain size of about 5–10 μm estimated from the optical microscope image. We suggest this change in texture corresponds to the N–SmZ_A_ transition. Importantly, in the SmZ_A_ the two distinct disclination lines remained at 9 and at 3 o'clock (Fig. 3, bottom), i.e., in the identical disclination configuration as in the smooth N-texture, despite the grainy microdomain features of the SmZA phase.

On further cooling at 1 K/min**,** the transition from SmZ_A_ to N_F_ occurred at *T* = 69 °C**,** at which the microdomain structure disappeared and a smooth texture with only one disclination was formed, characteristic of a polar N_F_ phase (left image of Fig. 3). However, reheating with a higher rate of 10 K/min from N_F_ into the SmZ_A_ phase (Fig. 3, top path) yielded an unexpected scenario. The single disclination line texture from the previous N_F_ phase was now maintained in the SmZ_A_ phase, giving the impression of a polar structure. On further heating at a rate of 0.5 K/min the singular disclination line then split up into two upon transitioning into the N phase at *T* = 83 °C cf. the upper path in Fig. 3.

**Figure 4:**
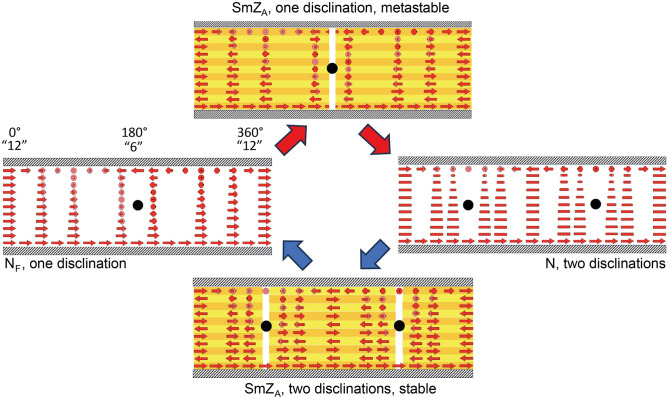
Proposed schematics of the structures of Fig. 3 in the case of planar signed layers, visualized through flattened-out, vertical cylindrical cuts through the CR cell (visualization of the cut found in the SI Video S2). The layers of the SmZ_A_ phase are indicated in yellow and orange, the disclination lines as black filled circles, and the polarization as red arrows. The white lines in the sketches of the SmZ_A_ phase indicate regions where the layer structure is unknown. However, it is important to note that crossing a disclination in the two disclination line case is accompanied by a change in layer count, leading to a dislocation. The SmZ_A_ state with one defect (top figure) is a metastable state, cf. Figure 6.

**Figure 5:**
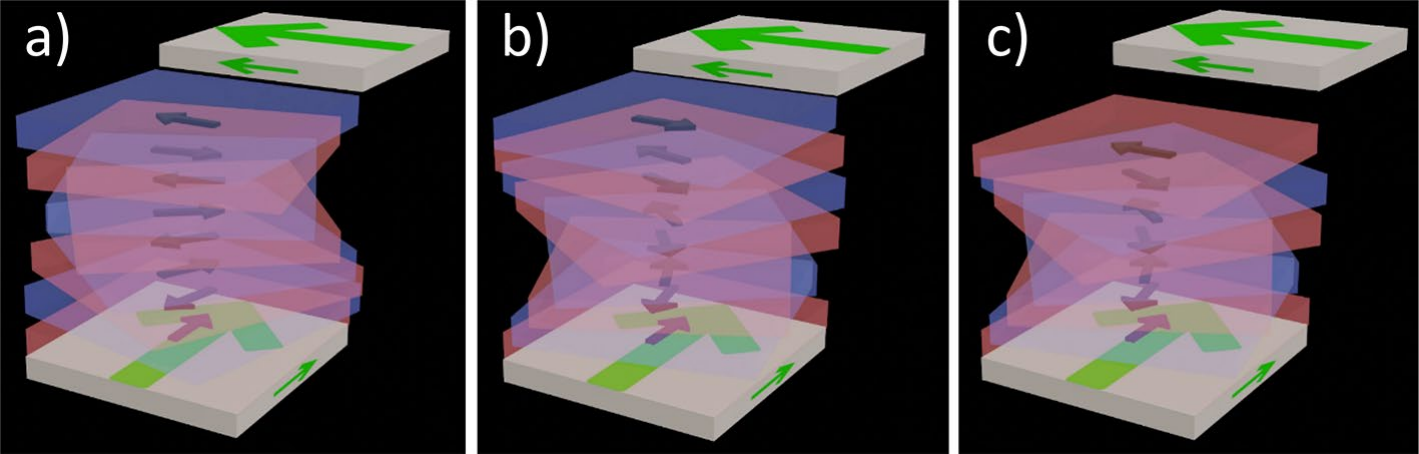
Handedness inversion of the twist at 3 and 9 o'clock in the SmZ_A_ phase. (**a**) shows a 90° twist that matches both boundary conditions imposed by the rubbing of the CR cell. Since a disclination line is present at 3 and 9 o'clock in the ground state (two disclination lines) of the texture, an inversion of the handedness must take place at 3 and 9 o'clock. Simply inverting the handedness (**b**) results in a mismatch and is therefore not viable. However, the phase can form one less layer (**c**), inverting the polarization vector by 180° and therefore matching the rubbing direction again.

To further understand the formed textures, one must consider the possible orientation of the director throughout the whole cell. In Fig. 4 vertical cylindrical cuts, coaxial with the circular rubbing are unrolled, so that on both ends of the cell the position 12 is seen (the cylindrical cut is visualized in SI Video S2). In between, positions 3, 6, and 9 o'clock are passed in that order, so that six is in the middle of the drawing. Furthermore, the different alignment cases, depending on the phase and history of the cell are depicted mimicking the positions of Fig. 3. In the case of the non-layered structures (N and N_F_), the director is free to twist, matching the local rubbing directions as previously described. However, the layered, antipolar, structure of the SmZ_A_ is more complex to match with the twisted alignment conditions. If the layers form parallel to the surface (horizontal configuration), a surface-induced twist does not impair the layering and is thus allowed. One could then speculate that the in-plane polarity of the boundary conditions come into play, which means that in the single disclination line SmZ_A_ case, the number of layers must be odd, so that the parallel rubbing direction at position 12 is satisfied by the polarization vectors. In principle the same number of layers can be kept in the whole CR cell, matching the local boundary conditions everywhere. However, in the two disclination line case, the antiparallel rubbing at position 6 and the lines at 3 and 9 force the number of layers to be changed to an even number, since an odd number of layers would mismatch one of the alignment conditions (cf. SI Video S3 and Fig. 5). But as the cell gap is 4 µm and one polar layer is about 10 nm thick, we argue this is not important; To match the boundary conditions, the decrease/increase in SmZ_A_ layer thickness would be only 0.3% resulting in a negligible change in twist elastic energy. In other words, the planar layer antiferroelectric structure of SmZ_A_ can always adapt to the (polar) boundary conditions, and the configuration with a minimum amount of necessary twist will form.

**Figure 6:**
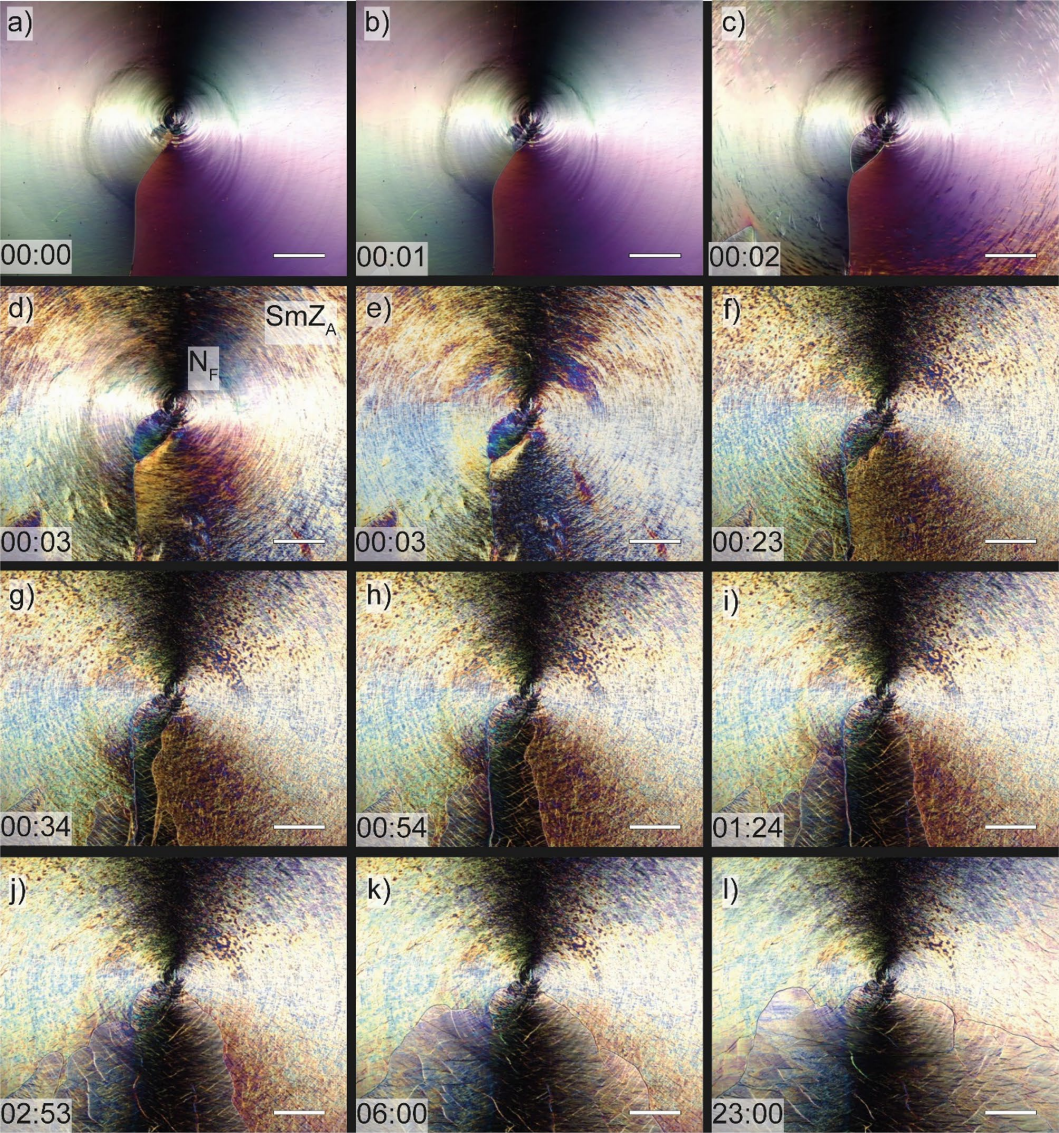
Textures of DIO in circularly rubbed 4 µm thick cells. (**a**) depicts the ferroelectric nematic phase with one single disclination line just below the transition into the SmZ_A_ phase. After heating above the transition temperature (**b**), the disclination line splits into two at the center of rubbing and some pinpoints out of frame, due to the loss of net polarization during this transition. However as seen in (**c**–**e**) the irregular texture forming from the outside inwards is the layered structure of the SmZ_A_ phase which keeps the single line from fully splitting, due to its increased viscosity. The transition from the metastable state with one disclination line to the stable state with two disclination lines now requires 23 h (**f**–**l**). All scale bars correspond to 200 µm.

An important observation is that when the sample is instead left in the SmZ_A_ after heating from the N_F_ phase, the initial single vertical disclination line at 9 o'clock slowly splits into two horizontal lines approaching 9 and 3 o'clock over a period of about 24 h (see Fig. 6). Considering the opposite approach via cooling, the initial two-disclination line texture is preserved over a longer period of time with no visible changes. This shows that the ground state of the SmZ_A_ is indeed the two-line configuration, while the one-line configuration is metastable. A similar metastable one defect line configuration in CR cells also might appear in conventional non-polar nematics, but only as a transient phenomenon, e.g. if they are electrically switched to the homeotropic state before the field is switched off. Then the nematic first relaxes into the one-line configuration, but immediately thereafter splits up in the stable two-line configuration, showing that the latter is the ground state, see SI Video S1. It is easy to understand why the two-line configuration has lower elastic energy than the on-line configuration. In the former, the twist ranges from 0 to ± 90° in both halves of the cell, while in the latter, one half of the cell has a twist from 0 to ± 90° while the other half has a higher twist, ranging from ± 90°to ± 180°. This makes it obvious, that the elastically favored ground state is the two disclination state. Hence, in DIO the one-line configuration in CR cells is inherited from the lower temperature N_F_ phase. We suggest that the slow dynamics of the relaxation, and the motion of the disclination lines, from the metastable one-line state to the two-line ground state is due to the high effective viscosity of the layered SmZ_A_ phase. Thus, there is no net ferroelectric polarization in the one-line metastable state of SmZ_A_; the rapid phase transition from ferroelectric N_F_ to antiferroelectric SmZ_A_, is followed by the much slower transition from the one-, to the two-line configuration. The latter is only a structural change, not a change in the polar order. The SmZ_A_ phase is always nonpolar.

Let us now focus on the irregularities of the SmZ_A_ texture, cf. Figs. 3, 6. It has been reported that the SmZ_A_ phase often display a less smooth, even referred to as “furry”^2^, texture than both the N and N_F_ phases. The fact that the SmZ_A_ can adopt either bookshelf or planar alignment of the layers for parallel and antiparallel rubbing of the polymer alignment layers^45^ shows that the in-plane polar anchoring tendency has little or no effect on the alignment of the macroscopically nonpolar SmZ_A_.

**Figure 7:**
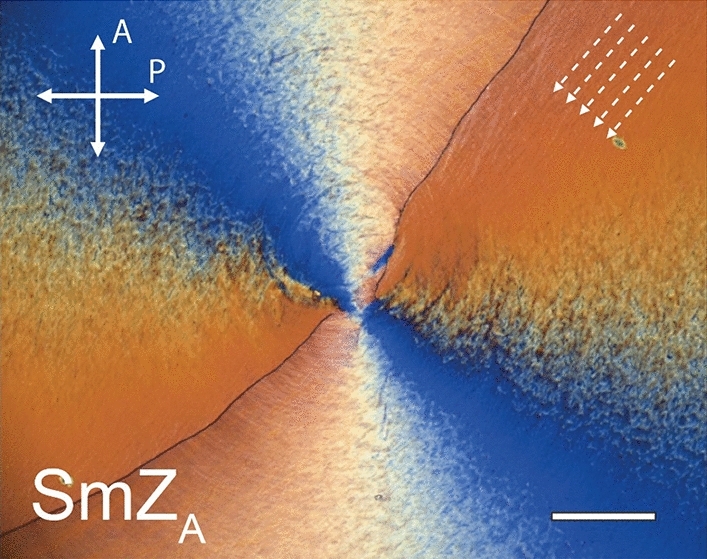
CR cell with DIO in the SmZ_A_ phase. The linear rubbing direction is from the upper right to lower left. For parallel/antiparallel rubbing a rather even texture with blue birefringence color is observed. This is compatible with a bookshelf, or a chevron structure. Further away from these regions the mutual rubbing directions do not allow for a bookshelf structure and the structure becomes grainy. Furthermore, it shifts in color and is fuzzy, reminding of a broken twisted structure. Even further away, the structure becomes orange and less irregular again and we suggest this corresponds to a planar layer structure, which is compatible with twist, as discussed in the text and shown in Fig. 4. Scale bar is 200 µm.

Figure 7 shows a 4 μm thick CR cell in the SmZ_A_ phase after cooling from the N phase. The cell is rotated so the linear rubbing direction makes about 45° with the horizontal direction, i.e., from lower left to upper right. In the regions where the circular and linear rubbing directions are essentially parallel or antiparallel (i.e. at 12 and at 6 o’clock) a well-aligned texture with blue birefringence color is obtained. In some CR cells we observed zig-zag-like defects in these regions (cf. SI Fig. S4), indicating the presence of a chevron-structure, as discussed in detail by Chen et al.^45^. We therefore suggest these regions approximately display a bookshelf or vertical chevron structure. Away from these regions the mutual rubbing directions deviate more and more from each other, and hence, the boundary conditions can no longer support a well-defined bookshelf structure. The material should now have two options; to break up in a broken twisted structure with azimuthally shifted blocks of SmZ_A_ (cf. SI Fig. S2), or to instead reorient to make the layers parallel to the surfaces, cf. Figure 4. At large angles between the two rubbing directions one could then expect a more irregular, domain-like texture, where the domain boundaries could be a result of a mix of horizontal layer regions and different amounts of domains with layers in the vertical direction, and/or broken twisted layer structures. In Fig. 6j–l, at 9 and at 3ʹ, a fuzzy, unsharp texture is observed, with a certain resemblance of smectic TGB textures. This could be an indication that broken twisted structures also occur in SmZ_A_, as one could speculate that both such broken twisted structures could have similar optical characteristics. If there is, at least locally, a twisted bookshelf SmZ_A_ structure one could at this stage only speculate regarding the structure in the boundaries between the azimuthally shifted SmZ_A_ blocks. One option could be that the material adopts a nematic (N) phase in the boundary regions to mediate a twist between blocks. An analogous case was in fact reported by Ruan et al.^49^, who studied twisted nematic cells undergoing the transition to smectic A on cooling. They found that below the N to smectic A transition the material SCE13 formed one or more blocks of uniform smectic A, separated by a twisted nematic region with two twisted nematic boundary regions. In addition to this “melted” twisted grain boundary model, one could contemplate a more complex SmZ_A_ structure, where the boundaries do not melt but instead contain a set of equally spaced dispirations, i.e., unit screw-dislocations, combined with half-unit disclinations. A similar structure has been proposed in relation to a hypothetical antiferroelectric TGB smectic C_a_* phase^50^.

In conclusion, based on CR cell experiments, we find that the SmZ_A_ phase is macroscopically non-polar, in agreement with the conclusions of Chen et al.^45^. At this stage our discussion about layer orientation in CR cells is somewhat speculative. Furthermore, we suggest that the SmZ_A_ phase may orient with standing and/or planar layers on rubbed polymer surfaces. Bookshelf alignment should be allowed for close to parallel and/or antiparallel rubbing. For non-parallel rubbing, planar layers should be preferred as this geometry allows for a defect free twist, even though an energetically more costly broken twisted configuration of SmZ_A_ blocks with vertical layers could be allowed in principle.

## Methods

### Materials

The liquid crystalline materials, DIO and AUUQU-2-N, were provided by Merck. The synthesis of DIO can be found for example in^8^. The synthesis of AUUQU-2-N is described in the patent DE 103 53 658 A1 2004 06 09. The phase sequence for both molecules is as follows:

AUUQU-2-N: Iso—128°C–N—83°C–SmZ_A_—69°C–N_F_—55°C–Cr

DIO: Iso—174°C–N—83°C–SmZ_A_—69°C–N_F_—34°C–Cr

Both are represented in cooling sequences, as the N_F_ and SmZ_A_ are monotropic in nature.

### Polarized optical microscopy

CR cells with cell gaps of 4 μm were filled with DIO, in the isotropic phase by means of capillary filling in the isotropic phase. Phase transition temperatures, textures and alignment were investigated with a Leica DM 2700 P polarized light microscope, equipped with an Instec heating stage HCS302 controlled by a mk1000 temperature unit. The pictures were taken by a pixeLink video camera. The videos were captured with the software OBS.

### Circularly rubbed cells

Clean ITO-coated three square inch sodalime glass plates of thickness 1.1 mm were spin-coated with polyimide PI2610 (DuPont) (5%) at 5000 rps for 30 s, and subsequently baked in an oven at 300° for 3 h. For linear rubbing, a commercial rubbing machine (LCTec Automation) was used. For circular rubbing we used a commercial, simple pillar drill, with a flat chuck covered with a piece of rubbing cloth (velvet). The rotating chuck was gently pressed onto the substrate for a few seconds. On each substrate we made 25 zones of circular rubbing, in a 5 × 5 array. UV-hardening glue containing 4 μm silica spacers was dispensed on the linearly rubbed plate using an in-house built computer-controlled dispenser. Thereafter, one linearly and one circularly rubbed substrate were assembled together using a commercial substrate assembling machine (Ciposa), and the glue was cured while pressing the substrates together. Each assembly was cut in 25 CR cells. The cells were capillary filled with liquid crystal in the isotropic phase. For the experiments reported here, we did not make use of the ITO electrodes.

### Supplementary Information

Supplementary Information.

Supplementary Video S1.

Supplementary Video S2.

Supplementary Video S3.

## Data Availability

The authors declare that the data supporting the findings of this study are available within the paper and its Supplementary Information files. Should any raw data files be needed in another format they are available from the corresponding author upon reasonable request.
