# Supplementary material for: Revealing the antipolar order in the antiferroelectric SmZA phase by means of circular alignment
Source: Sci Rep. 2024 Jul 1;14:15018. doi: 10.1038/s41598-024-65275-y (PMC11217385; doi:10.1038/s41598-024-65275-y)
Supplement: Supplementary file 1 — Supplementary Information. [file 41598_2024_65275_MOESM1_ESM.docx]

**Revealing the antipolar order in the antiferroelectric SmZ_A_ phase by means of circular alignment**

# Pierre Nacke^1^, Rachel Tuffin^2^, Melanie Klasen-Memmer^2^, Per Rudquist^3,**^, Frank Giesselmann^1,*^

^1^University of Stuttgart, Institute of Physical Chemistry, Stuttgart, 70569, Germany

^2^Merck Electronics KGaA, Display Solutions, Darmstadt, 64293, Germany

^3^Chalmers University of Technology, Department of Microtechnology and Nanoscience, Gothenburg, 41296, Sweden

[^*^frank.giesselmann@ipc.uni-stuttgart.de](mailto:*frank.giesselmann@ipc.uni-stuttgart.de)

[^**^per.rudquist@chalmers.se](mailto:**per.rudquist@chalmers.se)

Figure S1: Representation of possible layer orientations with different boundary conditions. (a) is a so-called bookshelf configuration where the layer normal is parallel to the surface of the cell. The alternating polarization would mismatch the alignment condition every second layer but since these layers are only about 9 nm in thickness, two of the layers can be seen as a non-polar block in a first approximation, interacting similarly to the paraelectric case. Parallel and antiparallel rubbing should give the same bookshelf structure. However, a bookshelf structure is incompatible with non-parallel (twisted) boundary conditions, which is inevitable in the circular rubbed cells moving off the 12 or 6 position, (b). To avoid twist deformation of the layers can instead lie flat with their layer normal perpendicular to the surface. Now only one layer interacts with the boundary condition, i.e. their polarization must match these conditions. This can be achieved by moving each layer slightly in a helical fashion, so that both the top and bottom conditions are matched. This has a crucial advantage: if the layers need to invert the handedness of the helix, this is easily done in this scenario (ref. Video SI3).


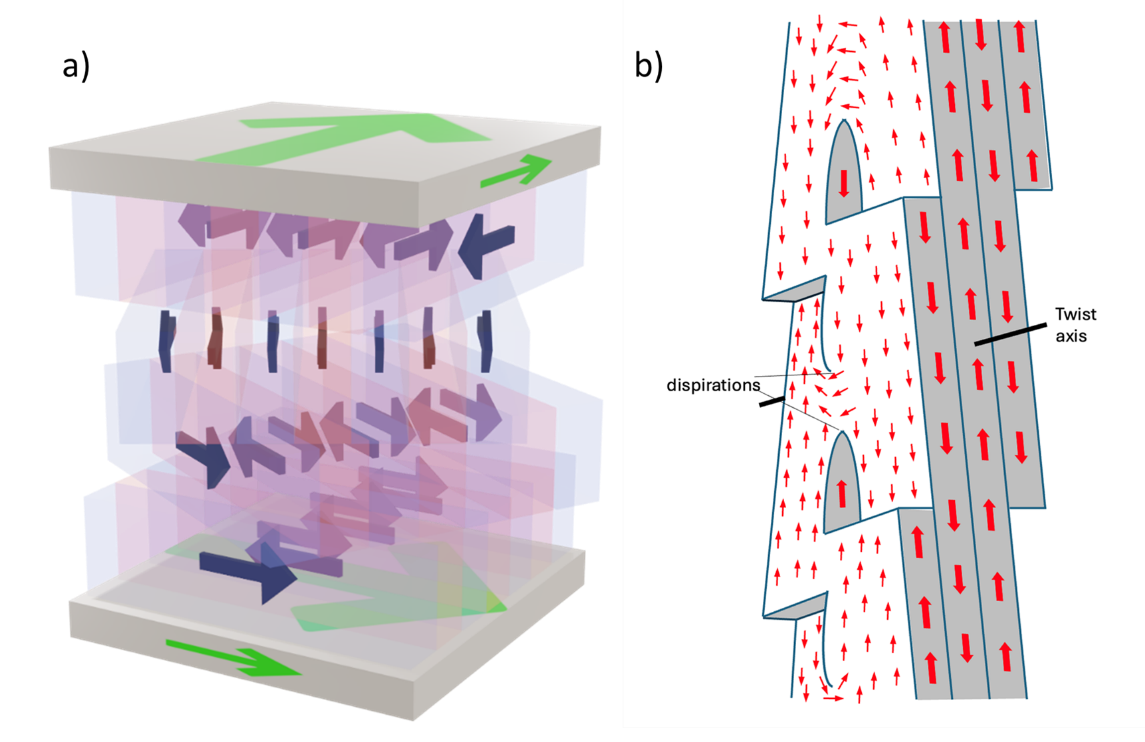


Figure S2: (a) Hypothetical schematic representation of the SmZ_A_ phase in a circularly rubbed (CR) cell with a twisted broken smectic structure. The polarization vector is depicted with black arrows. Each block of SmZ_A_ phase is slightly shifted with respect to each other to accommodate for the twist enforced by the rubbing direction of the alignment layer (green arrow). We speculate that the  boundary region between blocks could consist  of conventional nematic phase (“melted” boundary) or  a set of parallel unit screw dislocations combined with half unit disclinations, i.e. dispirations, (b). The latter would resemble  a twist grain boundary (TGB) - like smectic structure. The melted boundaries are a more straightforward explanation. Nonetheless, the dispiration hypothesis, although very speculative, in principle, would allow for a twisted, vertical layer structure as well.


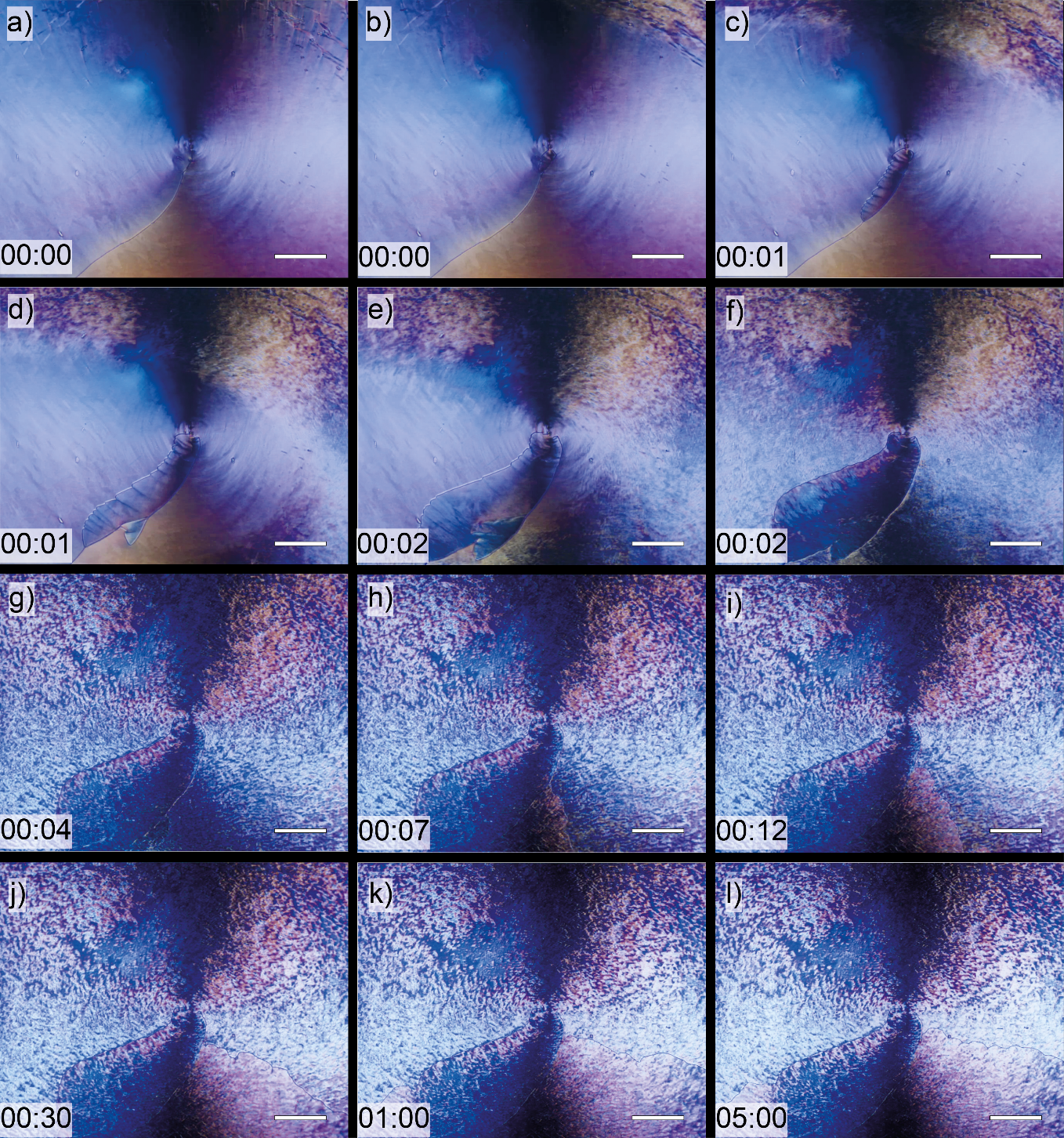


Figure S3: Long time observation of AUUQU-2-N after transitioning into the SmZ_A_ phase from the N_F_ phase. The same relaxation as in DIO into the two disclination ground state can be observed for AUUQU-2-N. All scale bars are 200 µm.

Figure S4: Depiction of the zig-zag defects sometimes found for DIO in the 12 and 6 position (a). The zig-zags appear when the layers kink from a bookshelf into a vertical chevron structure. Since the initial bookshelf structure can only appear when the rubbing directions are essentially parallel or antiparallel, the zig-zags are only observed in these two positions (cf. Figure S1). These can be compared to zig-zag defects of the same material in parallel (b) or antiparallel rubbed cells. (c) is a less magnified image of the zig-zags.

**Supplementary Videos Legend**

Video S1: Switching experiment with E7. The liquid crystal was filled inside the CR cell with a nematic, two disclination line texture observable. A square AC voltage of ~80V_pp_, 100 Hz, is applied normal to the cell, leading to a homeotropic alignment of the director **n**. After switching off the applied field, a single disclination line state can be seen. It slowly transitions to the original two-line ground state over a few seconds, opening up the disclination line from position 6.

Video S2: Illustration of the cylindrical cuts which are flattened out in Figure 4 in the article**.**

Video S3: Demonstration of mismatching layers during the inversion of the helix handedness. In CR cells, at the 3 and 9 position, a disclination line is formed, as a consequence of the change in twist handedness (twist inversion). However, after twist inversion the antipolar ordering of the layers would create a mismatch with one of the polar boundary conditions. This can be solved by the removal of one layer to re-establish the matching boundary conditions.
